# Author Correction: Optogenetic-controlled immunotherapeutic designer cells for post-surgical cancer immunotherapy

**DOI:** 10.1038/s41467-024-48084-9

**Published:** 2024-05-01

**Authors:** Yuanhuan Yu, Xin Wu, Meiyan Wang, Wenjing Liu, Li Zhang, Ying Zhang, Zhilin Hu, Xuantong Zhou, Wenzheng Jiang, Qiang Zou, Fengfeng Cai, Haifeng Ye

**Affiliations:** 1 Shanghai Frontiers Science Center of Genome Editing and Cell Therapy, Biomedical Synthetic Biology Research Center, Shanghai Key Laboratory of Regulatory Biology, Institute of Biomedical Sciences and School of Life Sciences, East China Normal University, Dongchuan Road 500, Shanghai, 200241 China; 2 Shanghai Institute of Immunology, Shanghai Jiao Tong University School of Medicine, 280 South Chongqing Road, Shanghai, 200025 China; 3 Department of Breast Surgery, Yangpu Hospital, School of Medicine, Tongji University, Shanghai, 200090 China

**Keywords:** Optogenetics, Cell therapies, Synthetic biology, Drug delivery

Correction to: *Nature Communications* 10.1038/s41467-022-33891-9, published online 26 October 2022

The original version of the manuscript contained errors in Fig. 3, in Supplementary Fig. 14, and in Source Data for Fig. 3c. Original Fig. 3 has now been replaced with the corrected version in the Article file (PDF and HTML versions) and an updated Supplementary Information file and Source Data file have been made available. In detail:

- In Fig. 3b, the image of one mouse (third from left) in the group “G5 FLICs-loaded hydrogel (FRL)” for Day 10 was incorrect and has been replaced with the correct image.
- The above applies also for Supplementary Fig. 14 that contains magnified images of the mice displayed in Fig. 3b.
- The correct bioluminescence value for the mouse above has been taken into account (information updated in the Source Data file for Fig. 3c) and Fig. 3c revised accordingly.

### Supplementary information

Updated Supplementary Information

Updated Source Data

