# Supplementary material for: Optogenetic-controlled immunotherapeutic designer cells for post-surgical cancer immunotherapy
Source: Nat Commun. 2022 Oct 26;13:6357. doi: 10.1038/s41467-022-33891-9 (PMC9605972; doi:10.1038/s41467-022-33891-9)
Supplement: Supplementary file 1 — Supplementary Information [file 41467_2022_33891_MOESM1_ESM.pdf]

Supplementary Information

Optogenetic-controlled immunotherapeutic designer cells for post-surgical cancer immunotherapy

Yuanhuan Yu[1#], Xin Wu[1#], Meiyan Wang[1#], Wenjing Liu[1], Li Zhang[1], Ying Zhang[1], Zhilin Hu[2], Xuantong Zhou[1], Wenzheng Jiang[1], Qiang Zou[2], Fengfeng Cai[3]* and Haifeng Ye[1]*

[1]Shanghai Frontiers Science Center of Genome Editing and Cell Therapy, Biomedical Synthetic Biology Research Center, Shanghai Key Laboratory of Regulatory Biology, Institute of Biomedical Sciences and School of Life Sciences, East China Normal University, Dongchuan Road 500, Shanghai 200241, China

[2]Shanghai Institute of Immunology, Shanghai Jiao Tong University School of Medicine, 280 South Chongqing Road, Shanghai 200025, China

[3]Department of Breast Surgery, Yangpu Hospital, School of Medicine, Tongji University, Shanghai 200090, China

[#]These authors contributed equally.

*To whom correspondence should be addressed: E-mail: caifengfeng@tongji.edu.cn and hfye@bio.ecnu.edu.cn

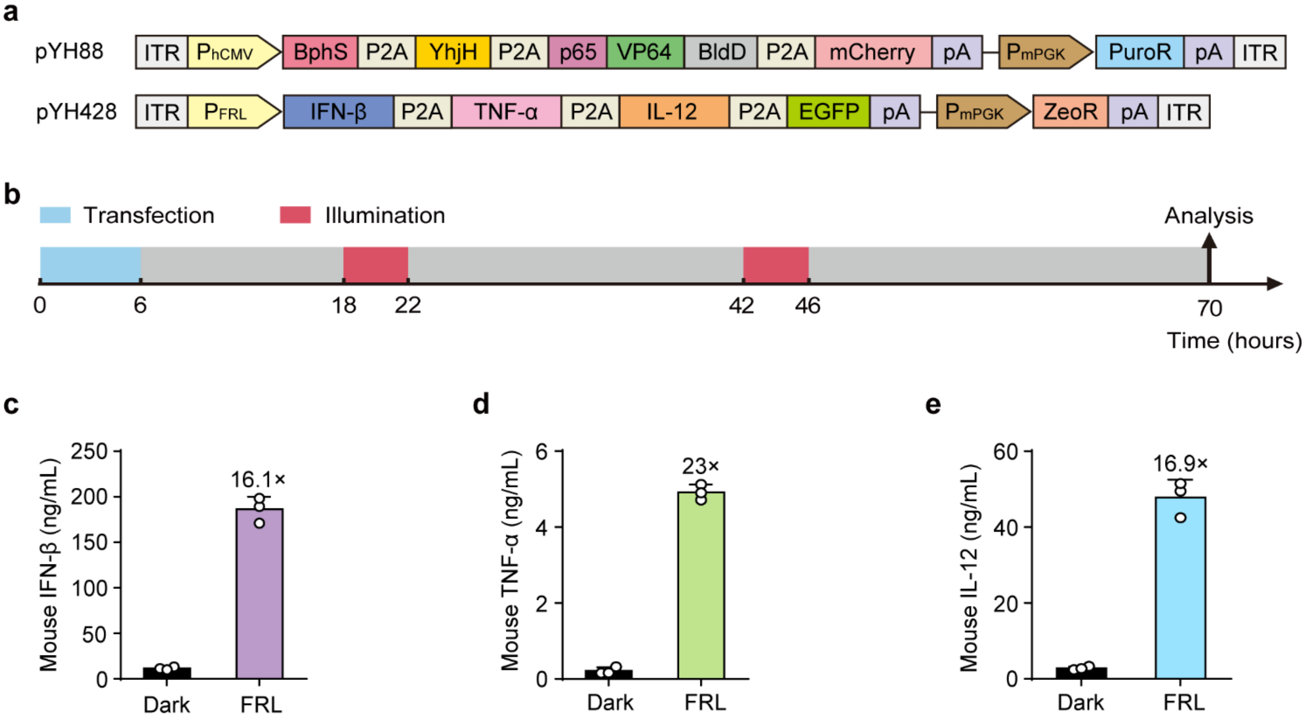

Supplementary Figure 1. The design and function test of the FRL-controlled cytokine secretion of FLICs. (**a**) Schematic depicting the genetic configuration of constructs used for FLICs containing FRL responsive module (pYH88, ITR-P_{hCMV}-BphS-P2A-YhjH-P2A-p65-VP64-BldD-P2A-mCherry-pA::P_{mPGK}-PuroR-pA-ITR) and the reporter vector (pYH428, ITR-P_{FRL}-IFN-β-P2A-TNF-α-P2A-IL-12-P2A-EGFP-pA::P_{mPGK}-ZeoR-pA-ITR). (**b**) Schematic diagram of the time schedule for FRL-controlled immunomodulatory cytokine secretion in hMSC-TERT cells. Transfected hMSC-TERT cells were illuminated (1 mW/cm^2; 730 nm) using a custom-designed 4 × 6 light-emitting diode (LED) array for 4 h once a day for 2 days and culture supernatant was scored using corresponding enzyme-linked immunosorbent assay (ELISA) kits 48 h after the first illumination. (**c-e**) FRL-controlled cytokines (IFN-β, TNF-α, and IL-12) release in hMSC-TERT cells. hMSC-TERT cells (6×10^4) were co-transfected with pYH88 (150 ng) and pYH428 (100 ng), and were illuminated with FRL (1 mW/cm^2; 730 nm) for 4 h once a day for 2 days, and culture supernatant was collected to quantify (c) IFN-β, (d) TNF-α and (e) IL-12 production using corresponding ELISA kits 48 h after the first illumination. Data in c to e are presented as the mean ± SD; n = 3 independent experiments. Source data are provided as a Source Data file.

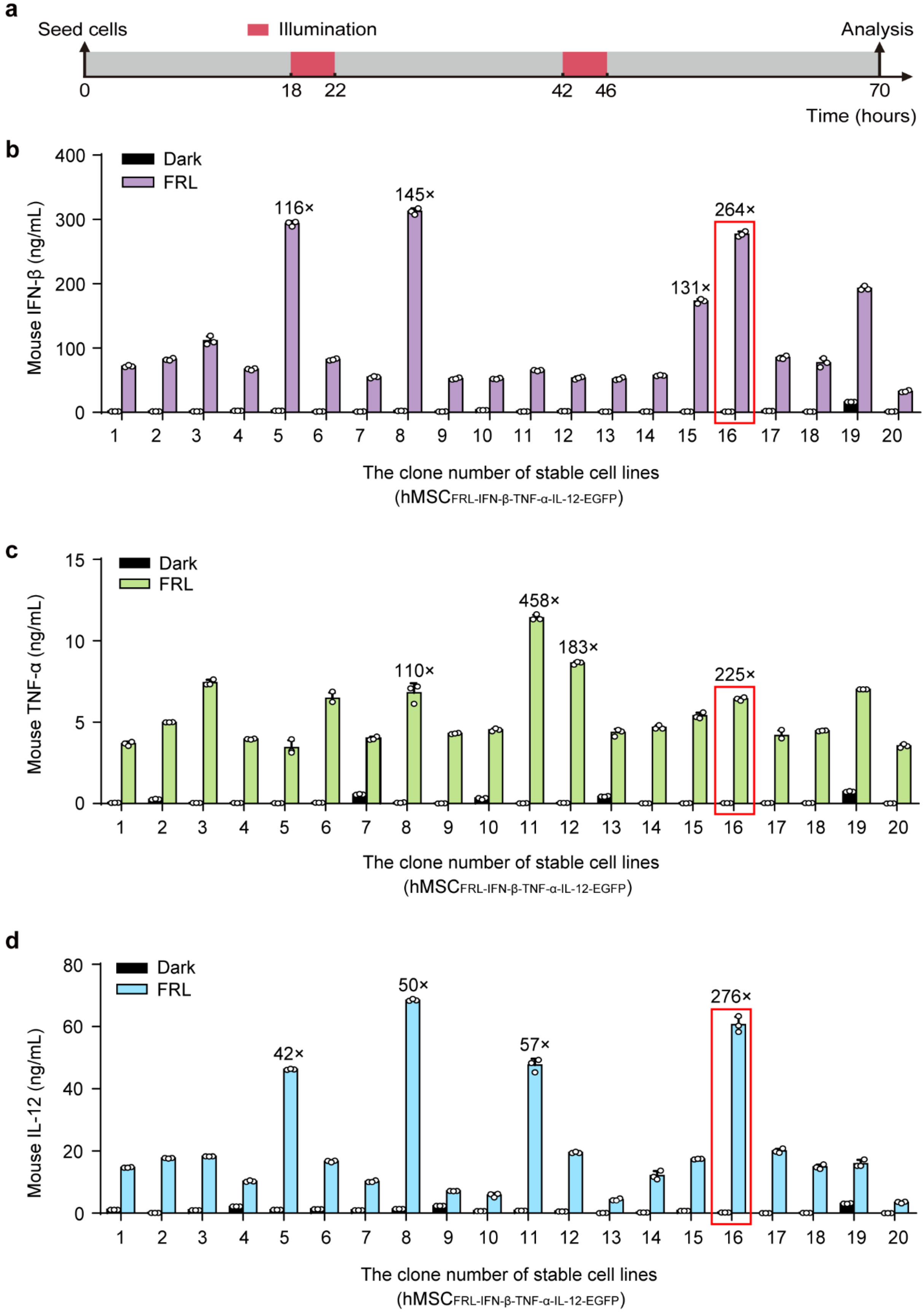

Supplementary Figure 2. Selection of stable FLIC lines. (**a**) Schematic diagram of the time schedule

for FRL-inducible immunomodulatory cytokine secretion in stable cell lines. The engineered stable $hMSC_{FRL\text{-}IFN\text{-}\beta\text{-}TNF\text{-}\alpha\text{-}IL\text{-}12\text{-}EGFP}$ were illuminated (1 mW/cm^2; 730 nm) with LED for 4 h once a day for 2 days and culture supernatant was scored using corresponding ELISA kits 48 h after the first illumination. (**b-d**) Construction and selection of stable cell lines. The selected cell clones were profiled for their FRL-inducible cytokines production performance. The hMSC-TERT cells were stably integrated with the light sensor module pYH88 and the light-responsive reporter module pYH428 using the Sleeping Beauty transposon system, and 20 randomly selected cell clones were profiled. Cytokines [(b) IFN-β, (c) TNF-α and (d) IL-12] production in the culture supernatant was scored 48 h after the first illumination. The red frame marks the best-in-class stable cell clone chosen for the following experiments. Data in b to d are presented as the mean ± SD; n = 3 independent experiments. Source data are provided as a Source Data file.

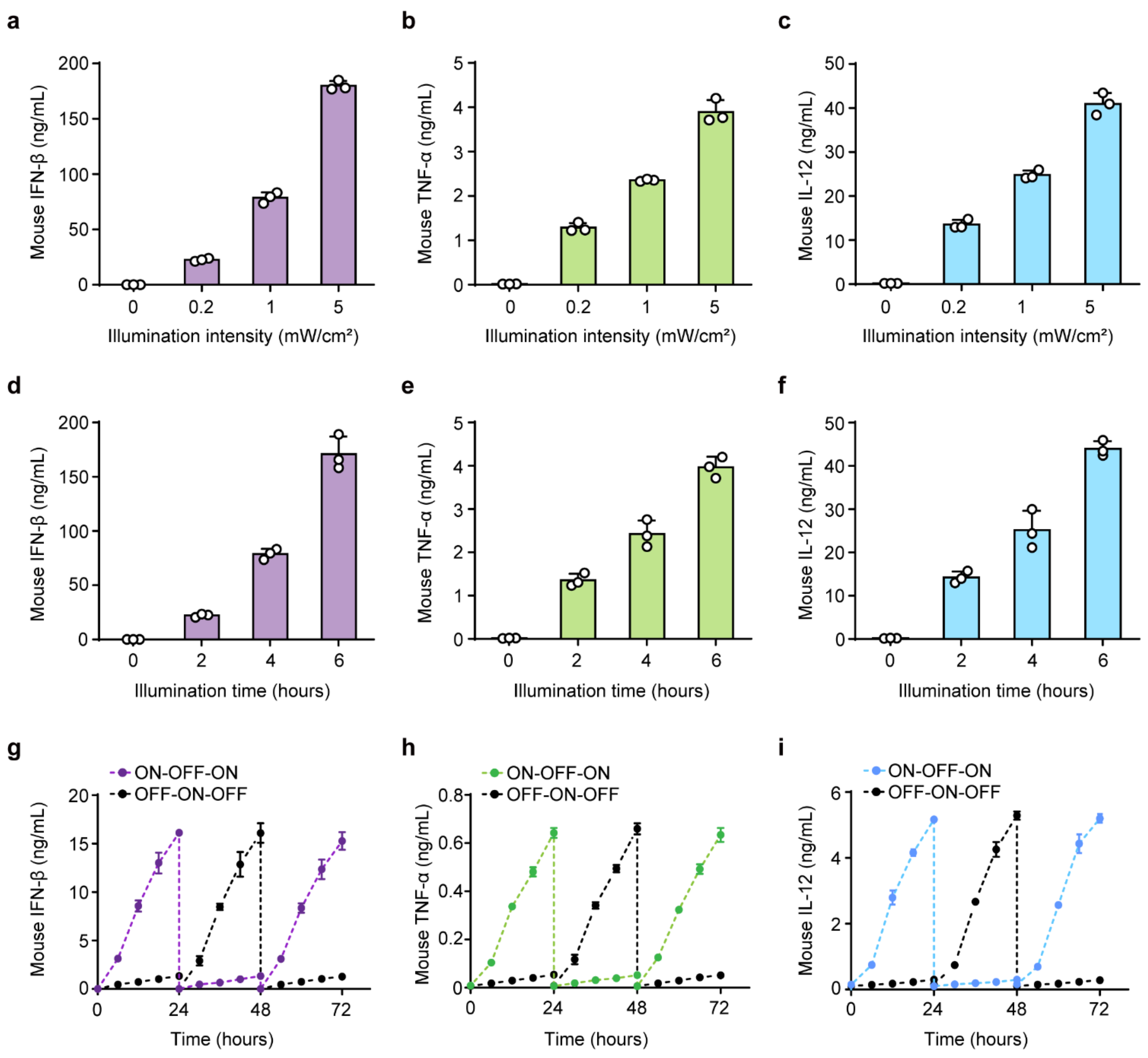

Supplementary Figure 3. Characterization of cytokine production kinetics of FLICs. (a-c) Illumination-intensity-dependent cytokines (IFN-β, TNF-α, and IL-12) release kinetics of FLICs. FLICs (3×10^4) were illuminated with FRL at different light intensities (0, 0.2, 1, 5 mW/cm^2) for 4 h once a day for 2 days, and culture supernatant was collected to quantify (a) IFN-β, (b) TNF-α, and (c) IL-12 production using corresponding ELISA kits 48 h after the first illumination. **(d-f)** Exposure-time-dependent cytokines (IFN-β, TNF-α, and IL-12) release kinetics of FLICs. FLICs (3×10^4) were illuminated with FRL (1 mW/cm^2; 730 nm) for 0, 2, 4, 6 h once a day for 2 days, culture supernatant was collected to quantify (d) IFN-β, (e) TNF-α and (f) IL-12 production using corresponding ELISA kits 48 h after the first illumination. **(g-i)** Reversibility of cytokines (IFN-β, TNF-α, and IL-12) release

of FLICs. FLICs were either kept in the dark (OFF) or illuminated with FRL (1 mW/cm^2) for 20 min (ON), and cytokines (g) IFN-β, (h) TNF-α, and (i) IL-12 production was scored every 6 h for 72 h. The culture medium was exchanged every 24 h. All data are presented as the mean ± SD; n = 3 independent experiments. Source data are provided as a Source Data file.

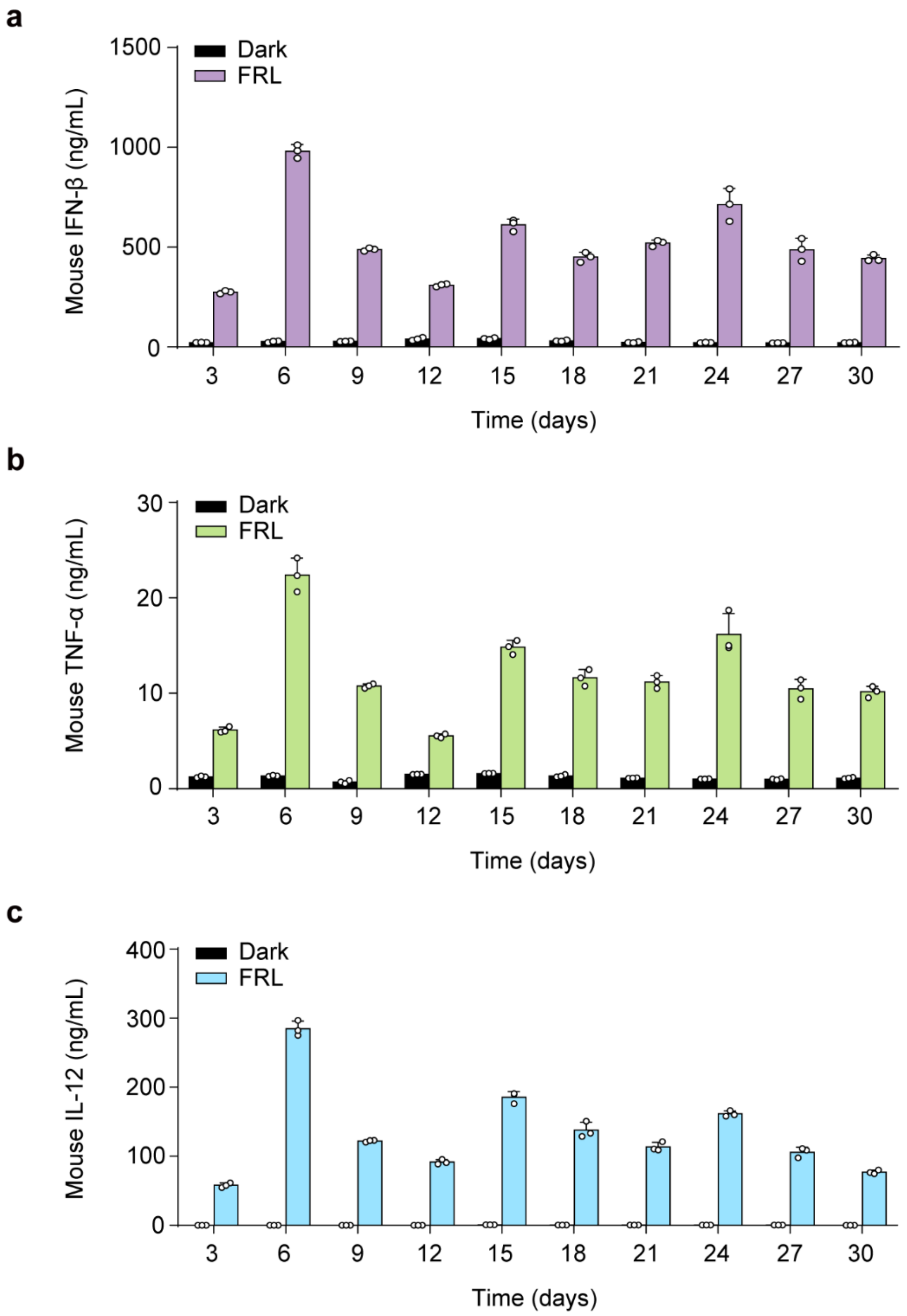

Supplementary Figure 4. Long-term performance of FRL-controlled cytokine production from FLICs-loaded hydrogel implants. (a-c) FLICs-loaded hydrogel implants (containing 2.5×10^6 FLICs) were solidified in a 48-well plate and illuminated with FRL (1 mW/cm^2; 730 nm) for 4 h once a day for 30 days. Culture supernatants were collected to quantify (a) IFN-β, (b) TNF-α and (c) IL-12 production using corresponding ELISA kits every three days. The cell culture medium was changed every three days accordingly. All data are presented as the mean ± SD; $n = 3$ independent experiments. Source data are provided as a Source Data file.

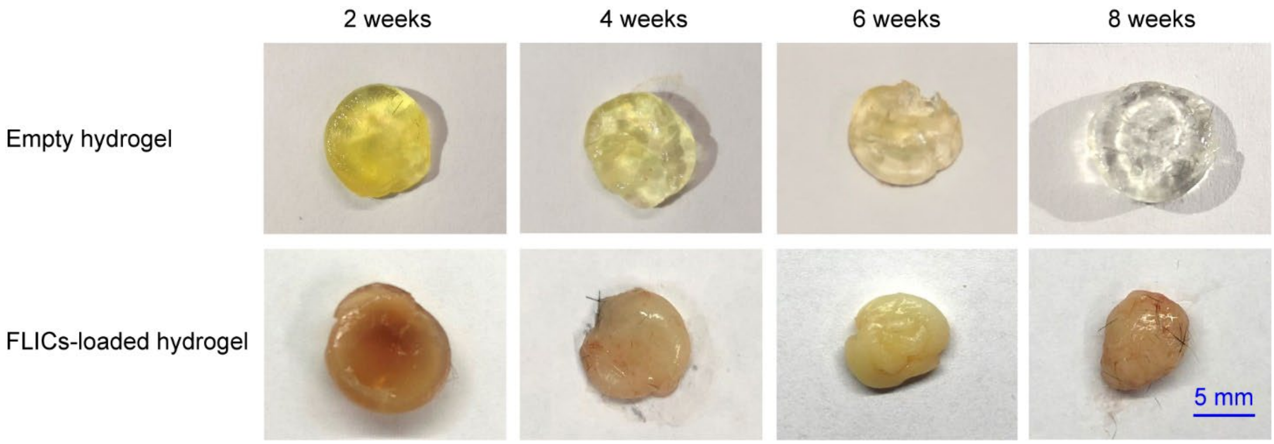

Supplementary Figure 5. Photographs of the hydrogel implants. Photographs showing representative hydrogel implants loaded with or without FLICs after implantation in mice at the indicated time points.

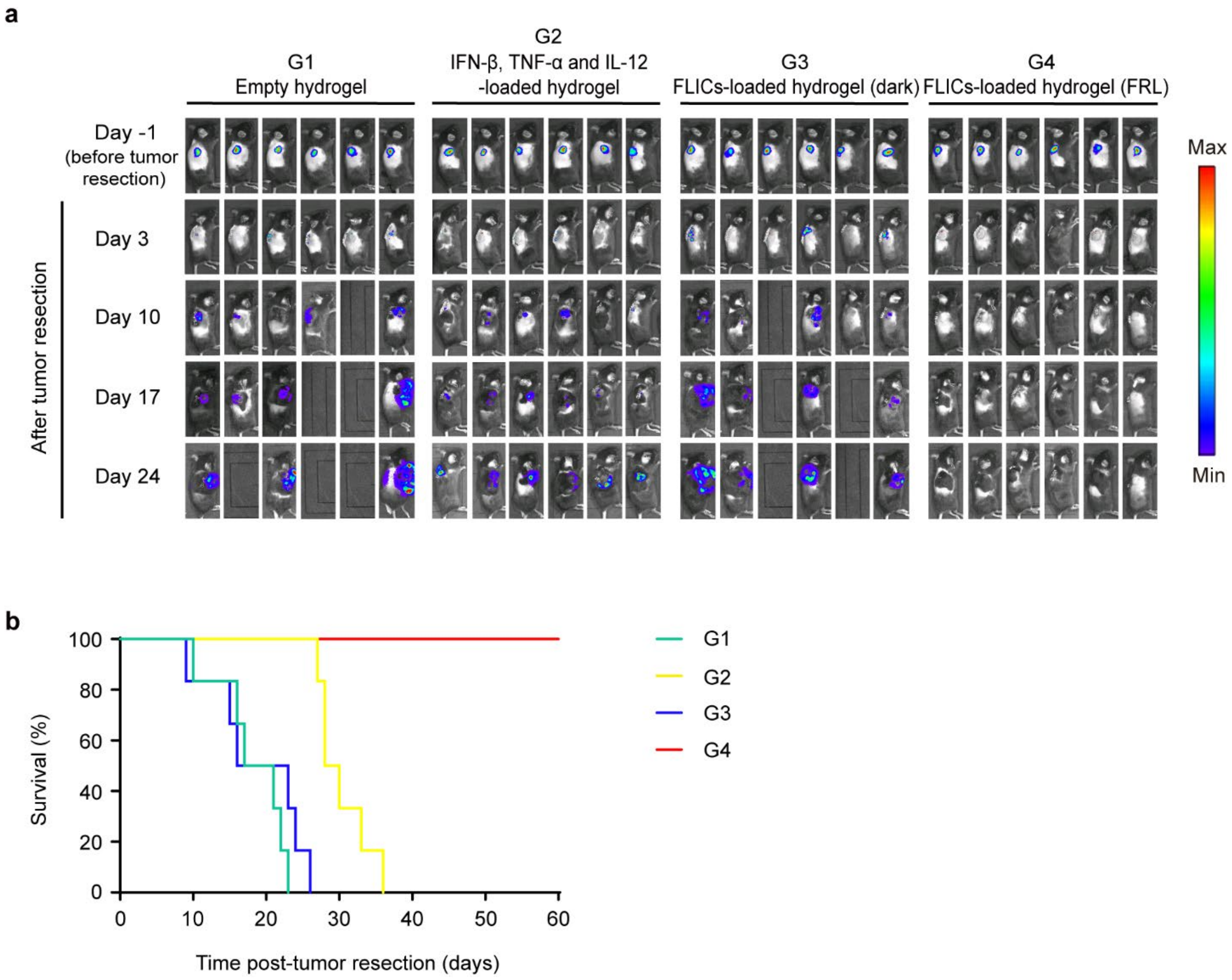

Supplementary Figure 6. Optogenetic control of cytokine release from FLICs-loaded hydrogel implants prevents tumor recurrence. Serial *in vivo* bioluminescence imaging of B16F10$_{Luc}$ tumors expressing luciferase before surgery and after surgical resection of primary tumors following resection site implantation of hydrogel scaffolds loaded with FLICs (IFN-β, TNF-α, and IL-12), or the recombinant cytokine proteins (IFN-β, TNF-α, and IL-12), or empty hydrogel scaffolds. (**a**) The mice were illuminated with FRL (10 mW/cm^2; 730 nm) for 2 h each day for 7 days. Mice implanted with empty hydrogels, or mice bearing the recombinant cytokine protein-loaded hydrogel implants or FLICs-loaded hydrogel implants but not exposed to FRL illumination were examined as controls. Six mice per group are shown. (**b**) Kaplan-Meier curves for mouse survival (n = 6 mice). Source data are provided as a Source Data file.

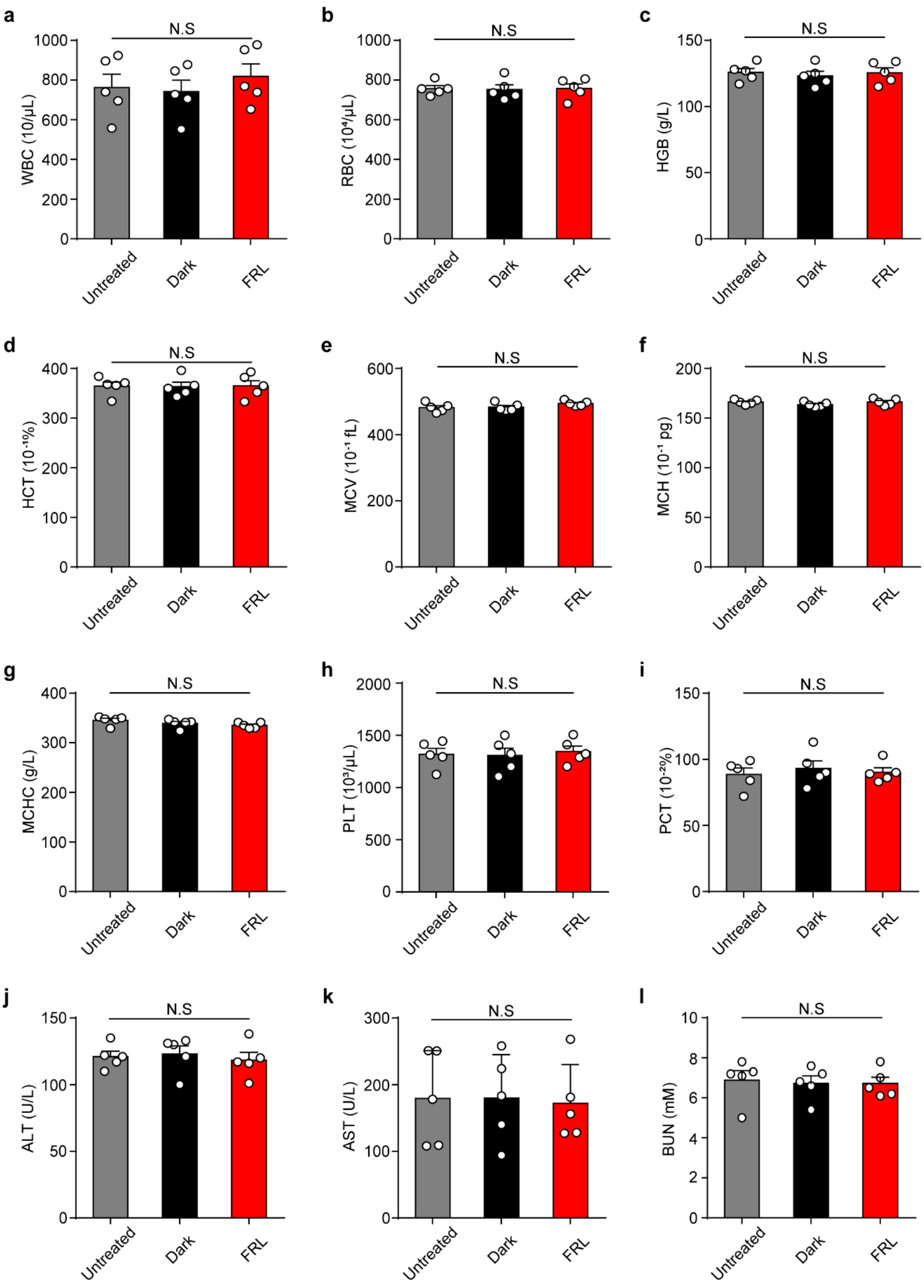

Supplementary Figure 7. Blood biochemistry and hematology analysis of mice implanted with FLICs-loaded hydrogel implants with or without FRL illumination or untreated healthy mice. Blood was recovered on day 14 from untreated healthy mice and the treated (with tumor resection)

mice with or without FRL illumination. (**a**) WBC, white blood cells; (**b**) RBC, red blood cells; (**c**) HGB, hemoglobin; (**d**) HCT, hematocrit; (**e**) MCV, mean corpuscular volume; (**f**) MCH, mean corpuscular hemoglobin; (**g**) MCHC, mean corpuscular hemoglobin concentration; (**h**) PLT, platelets; (**i**) PCT, platelet crit were tested using Sysmex XT-2000i automated haematology analyzer. (**j**) ALT, alanine aminotransferase; (**k**) AST, aspartate aminotransferase; (**l**) BUN, blood urea nitrogen was tested using corresponding ELISA kits. All data are presented as mean ± SEM ($n = 5$). P values were calculated by two-tailed unpaired t-test. *$P < 0.05$, **$P < 0.01$, ***$P < 0.001$, ****$P < 0.0001$. N.S, no significance. Source data are provided as a Source Data file.

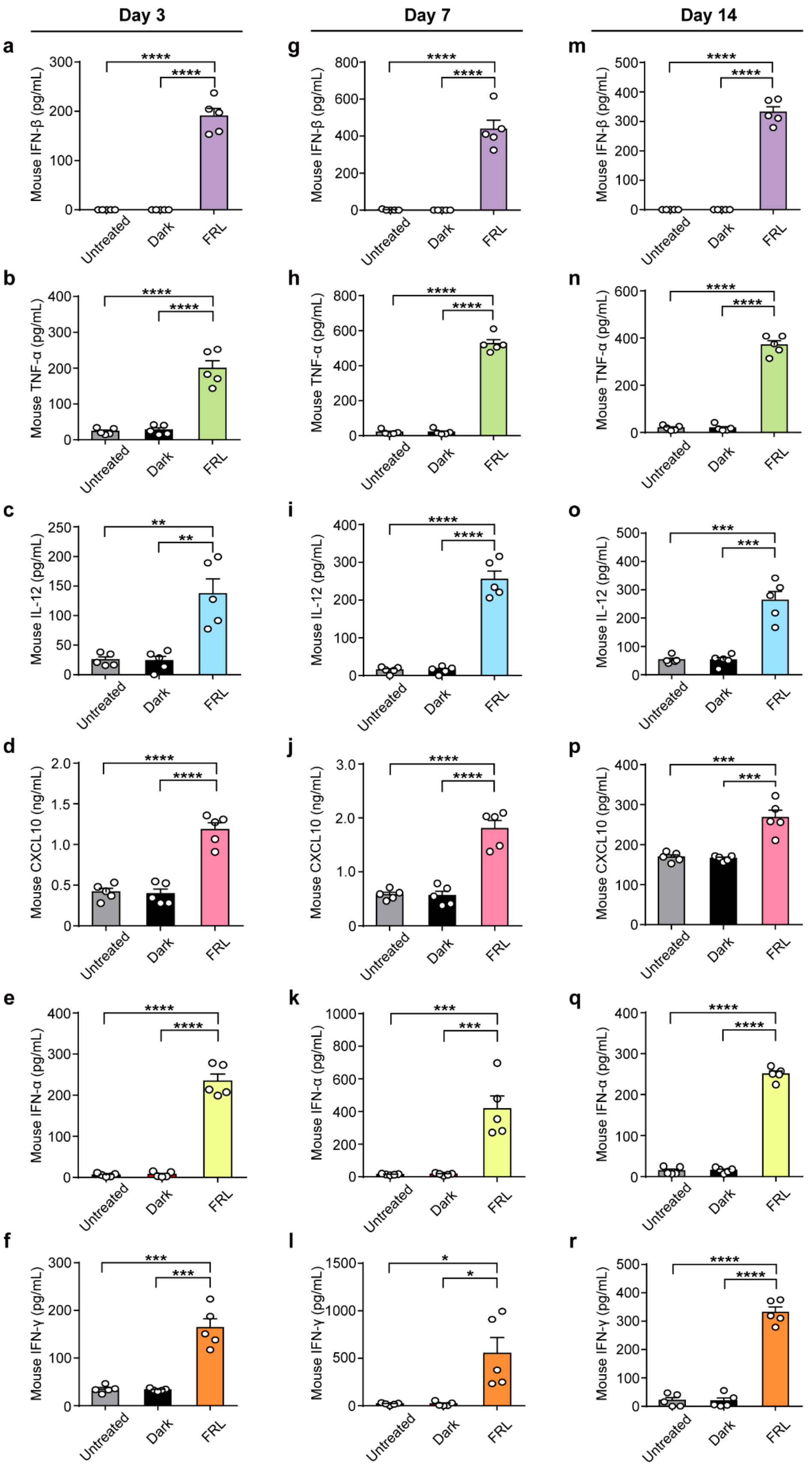

Supplementary Figure 8. Anti-tumor immune response (induced cytokines) mediated by FLICs-

loaded hydrogel implants. Tumors were resected from C57BL/6J mice 7 days after subcutaneous inoculation of B16F10$_{Luc}$ melanoma cells, and then FLICs-loaded hydrogel implants were surgically placed at the tumor resection site. Mice were illuminated with FRL (10 mW/cm^2; 730 nm) for 2 h each day for 14 days; Control mice groups included mice not given FRL illumination and mice that were not given a post-resection implant. Mouse plasma was collected on day 3, day 7, and day 14 after implantation for cytokine production analysis. (**a-r**) IFN-β, TNF-α, IL-12, CXCL10, IFN-α, and IFN-γ production in mice. The induced cytokines were quantified on day 3 (a-f), day 7 (g-l), and day 14 (m-r) using multiplexing laser bead-based immunoassay kits. a, ****P < 0.0001. b, ****P < 0.0001. c, **P=0.0022 and **P=0.0024, top to bottom. d, ****P < 0.0001. e, ****P < 0.0001. f, ***P=0.0002 and ***P=0.0001, top to bottom. g, ****P < 0.0001. h, ****P < 0.0001. i, ****P < 0.0001. j, ****P < 0.0001. k, ***P=0.00097 and ***P=0.00099, top to bottom. l, *P=0.0121 and *P=0.0122, top to bottom. m, ****P < 0.0001. n, ****P < 0.0001. o, ***P=0.0002 and ***P=0.0002, top to bottom. p, ***P=0.0009 and ***P=0.0006, top to bottom. q, ****P < 0.0001. r, ****P < 0.0001. All data are presented as the mean ± SEM (n = 5). P values were calculated by two-tailed unpaired t-test. *P < 0.05, **P < 0.01, ***P < 0.001, ****P < 0.0001. N.S, no significance. Source data are provided as a Source Data file.

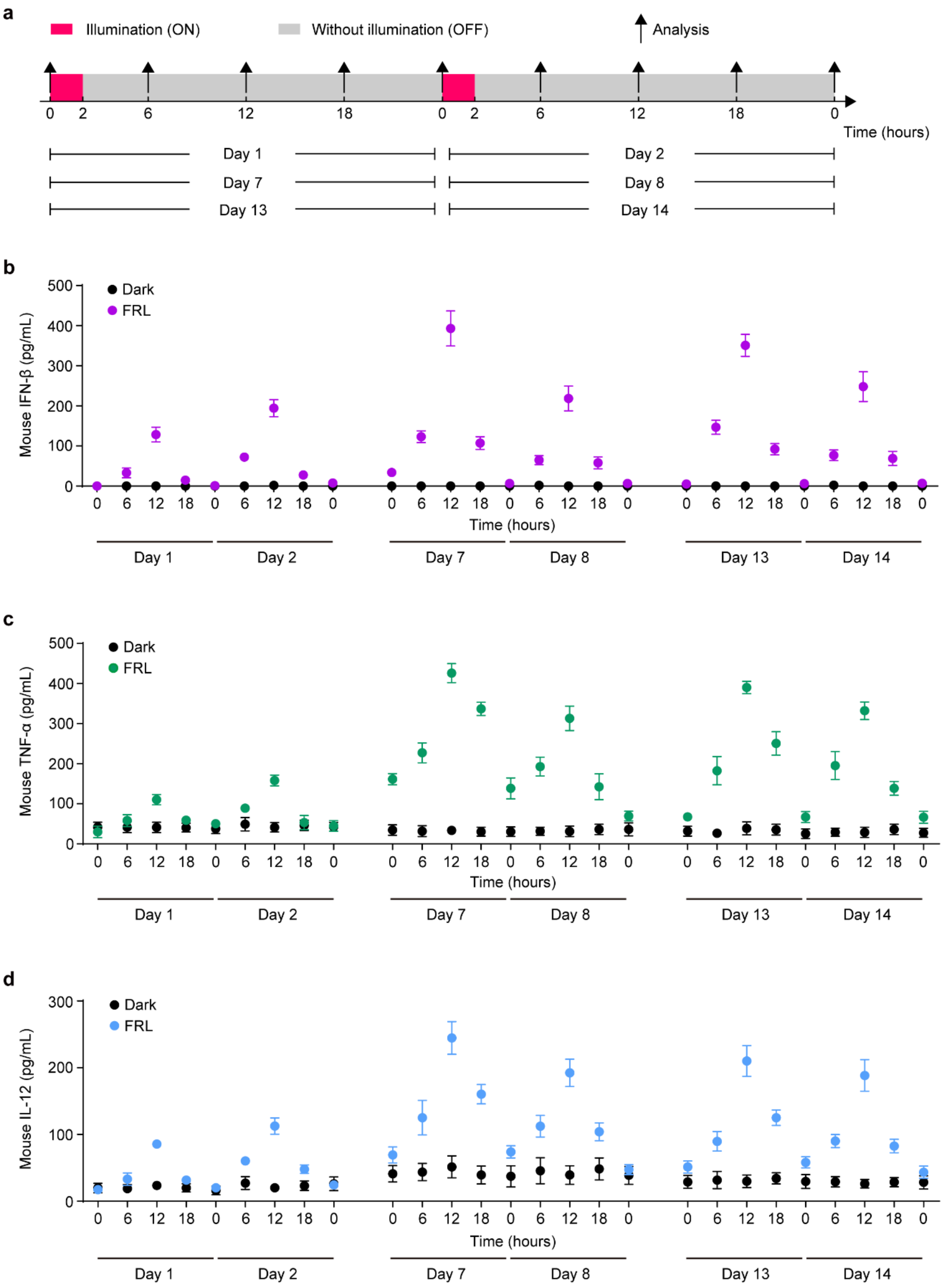

Supplementary Figure 9. The tunability of cytokines (IFN-β, TNF-α, and IL-12) release mediated by the FLICs-loaded hydrogel implants in mice. Tumors were resected from C57BL/6J mice 7 days after subcutaneous inoculation of B16F10$_{Luc}$ melanoma cells, and then FLICs-loaded hydrogel implants were surgically placed at the tumor resection site. (**a**) Schematic representation of the

experimental procedure and the time schedule used for evaluating cytokine secretion kinetics in mice. (**b-d**) Mice given FLICs-loaded hydrogel implants were illuminated with FRL (1 mW/cm^2; 730 nm) for 2 h (ON) and without FRL for 22 h (OFF) every 24 h for 14 days; control mice were not given FRL illumination (dark). Cytokines (IFN-β, TNF-α, and IL-12) production was quantified every 6 h on day 1, 2, 7, 8, 13 and 14 using LEGENDplex™ Multiplex Assay Kits. All data are presented as the mean ± SEM (n = 4). Source data are provided as a Source Data file.

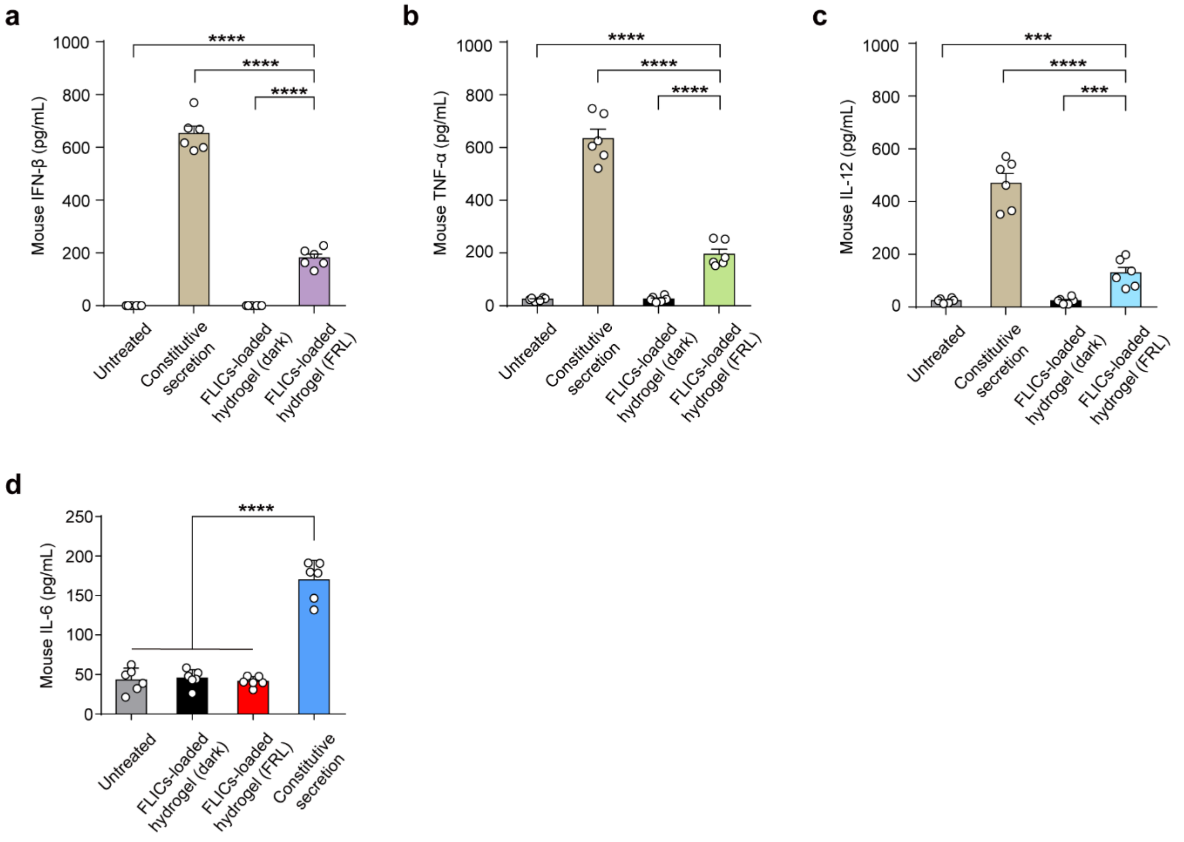

Supplementary Figure 10. Induced cytokines and IL-6 production in mice harboring the hydrogel implants loaded with FLICs or cells constitutively expressing the cytokines. Tumors were resected from C57BL/6J mice 7 days after subcutaneous inoculation of B16F10 $_{Luc}$ melanoma cells, and the hydrogel implants loaded with FLICs or cells constitutively expressing the cytokines (IFN-β, TNF-α, and IL-12) were surgically placed at the tumor resection site. Mice were illuminated with FRL (10 mW/cm^2; 730 nm) for 2 h each day for 3 days; control groups included mice given the hydrogel implants loaded with cells constitutively expressing the cytokines, mice given the hydrogel implants loaded with FLICs but not given FRL illumination, and mice that were not given a post-resection implant (untreated). Mouse plasma was collected on day 3 after implantation for cytokine production analysis. IFN-β (a), TNF-α (b), IL-12 (c) and IL-6 (d) were quantified on day 3 using multiplexing laser bead-based immunoassay kits. a, ****P < 0.0001. b, ****P < 0.0001. c, ***P=0.00069, ****P < 0.0001, ***P=0.00073, top to bottom. d, ****P < 0.0001. All data are presented as the mean ± SEM (n = 6). P values were calculated by two-tailed unpaired t-test. ****P < 0.0001, ***P < 0.001. Source data are provided as a Source Data file.

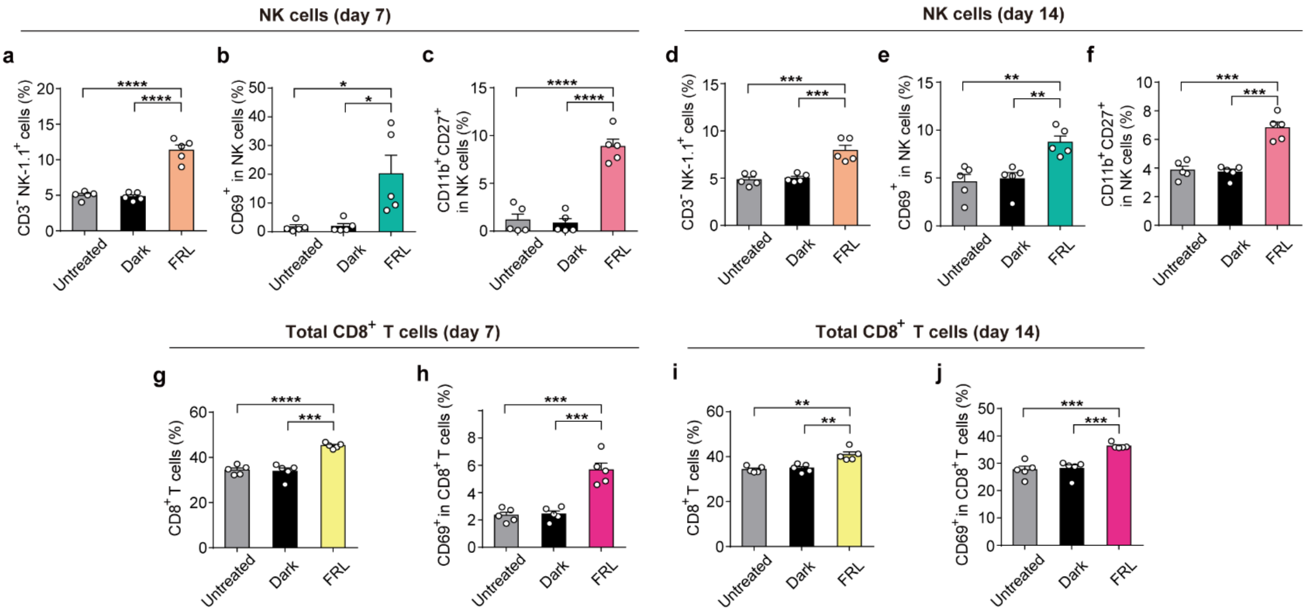

Supplementary Figure 11. Anti-tumor immune response (immune cells) mediated by FLICs-loaded hydrogel implants. (**a-c**) The activated and effector phenotypes of NK cells were evaluated in peripheral blood of mice sacrificed on day 7 by flow cytometry. Corresponding quantification of (a) NK cells, (b) CD69$^+$ NK cells, and (c) CD11b$^+$CD27$^+$ NK cells. a, ****$P < 0.0001$. b, *P=0.0223 and *P=0.0239, top to bottom. c, ****$P < 0.0001$. (**d-f**) Corresponding quantification of (d) NK cells, (e) CD69$^+$ NK cells, and (f) CD11b$^+$CD27$^+$ NK cells in peripheral blood of mice sacrificed on day 14. d, ***P=0.00098 and ***P=0.00096, top to bottom. e, **P=0.0036 and **P=0.0033, top to bottom. f, ***P=0.00029 and ***P=0.00012, top to bottom. (**g-h**) Corresponding quantification of (g) CD8$^+$ T cells and (h) CD69$^+$CD8$^+$ T cells in peripheral blood of mice sacrificed on day 7. g, ****$P < 0.0001$ and ***P=0.00012, top to bottom. h, ***P=0.00028 and ***P=0.00033, top to bottom. (**i-j**) Corresponding quantification of (i) CD8$^+$ T cells and (j) CD69$^+$CD8$^+$ T cells in peripheral blood of mice sacrificed on day 14. i, **P=0.0013 and **P=0.0034, top to bottom. j, ***P=0.00028 and ***P=0.00040, top to bottom. All data are presented as the mean ± SEM (n = 5 mice). P values were calculated by two-tailed unpaired t-test. *$P < 0.05$, **$P < 0.01$, ***$P < 0.001$, ****$P < 0.0001$. Source data are provided as a Source Data file.

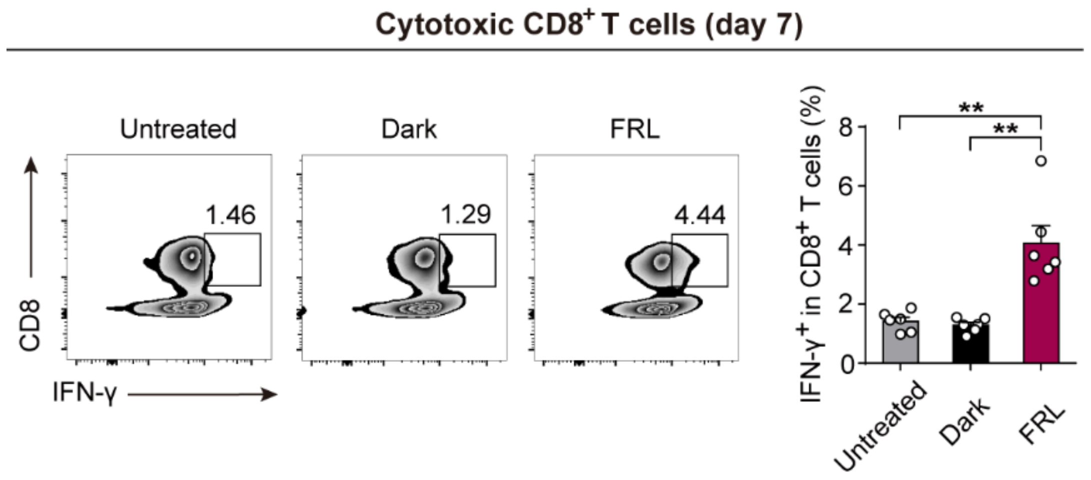

Supplementary Figure 12. Antigen-specific T cell response against tumor recurrence mediated by FLICs-loaded hydrogel implants. Tumors were resected from C57BL/6J mice 7 days after subcutaneous inoculation of B16F10-OVA (ovalbumin) melanoma cells, and FLICs-loaded hydrogel implants were surgically placed at the tumor resection site. Mice were illuminated with FRL (10 mW/cm^2; 730 nm) for 2 h each day for 7 days; control mice were not given FRL illumination (dark) and mice that were not given a post-resection implant (untreated). Spleens were removed and ground to collect lymphocytes on day 7 after implantation. Splenocytes were cultured with $OVA_{257\text{-}264}$ peptide (10 μg/mL). After 12 h, IFN-γ^+CD8$^+$ T cells were analyzed using flow cytometry. Representative flow cytometric analysis images (left) and corresponding quantification (right) of IFN-γ^+CD8$^+$ T cells in spleens of mice sacrificed on day 7. Data are presented as the mean ± SEM (n = 6 mice) except representative flow cytometric analysis images. P values were calculated by two-tailed unpaired t-test. **P=0.0017 and **P=0.0011, top to bottom. Source data are provided as a Source Data file.

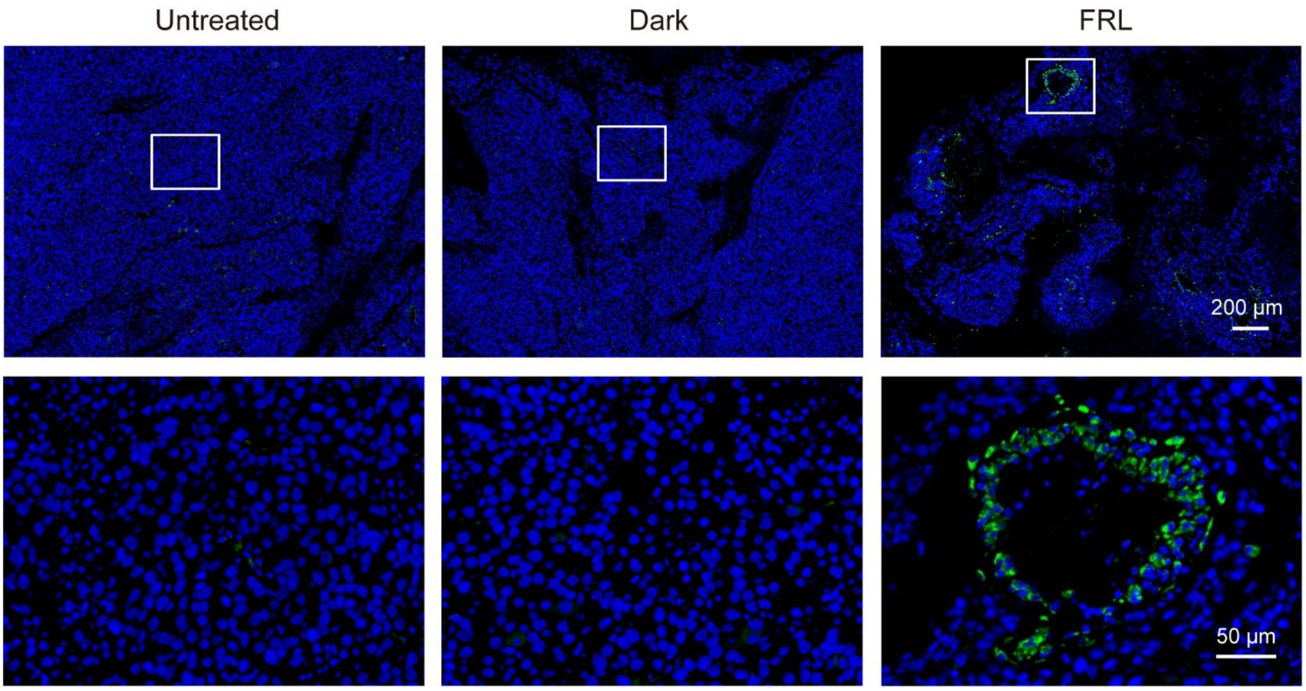

Supplementary Figure 13. Representative fluorescence microscopy images of CD8$^+$ T cells in tumor tissue slices. Microphotographs of representative examples of validation from immunofluorescence tumor infiltration CD8$^+$ T cells present in tumor paraffin sections from different treatment groups at 20 days after tumor resection. Blue for DAPI staining and green for CD8$^+$ T cells staining; White frames (top panels) and high magnification images (bottom panels) indicate details of local sites. The images represent typical results from three independent measurements (n = 5 mice). Scale bar, 200 μm (top panels); 50 μm (bottom panels).

Supplementary Figure 14. Magnified images of Figure 3b.

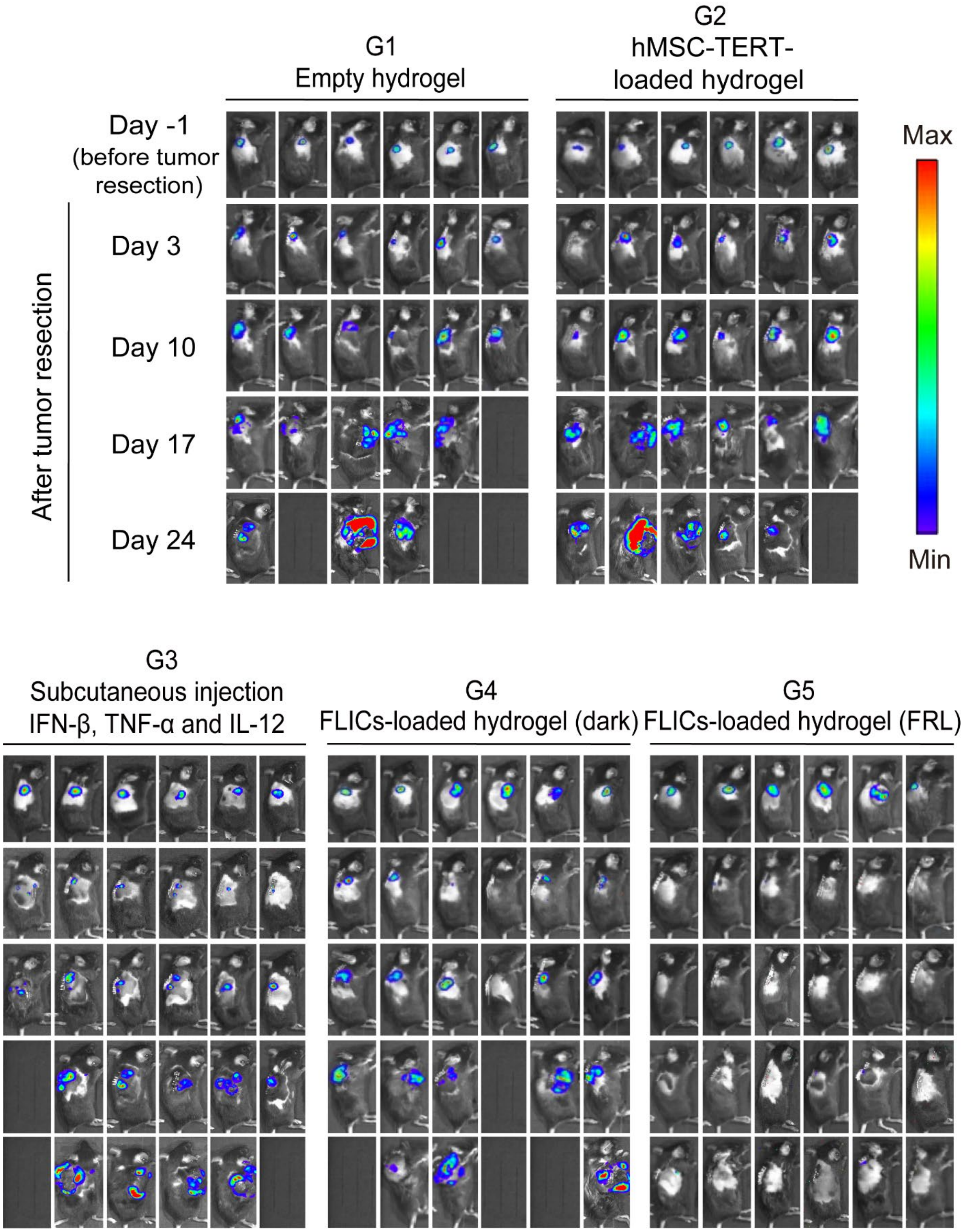

Supplementary Figure 15. Magnified images of Figure 4l.

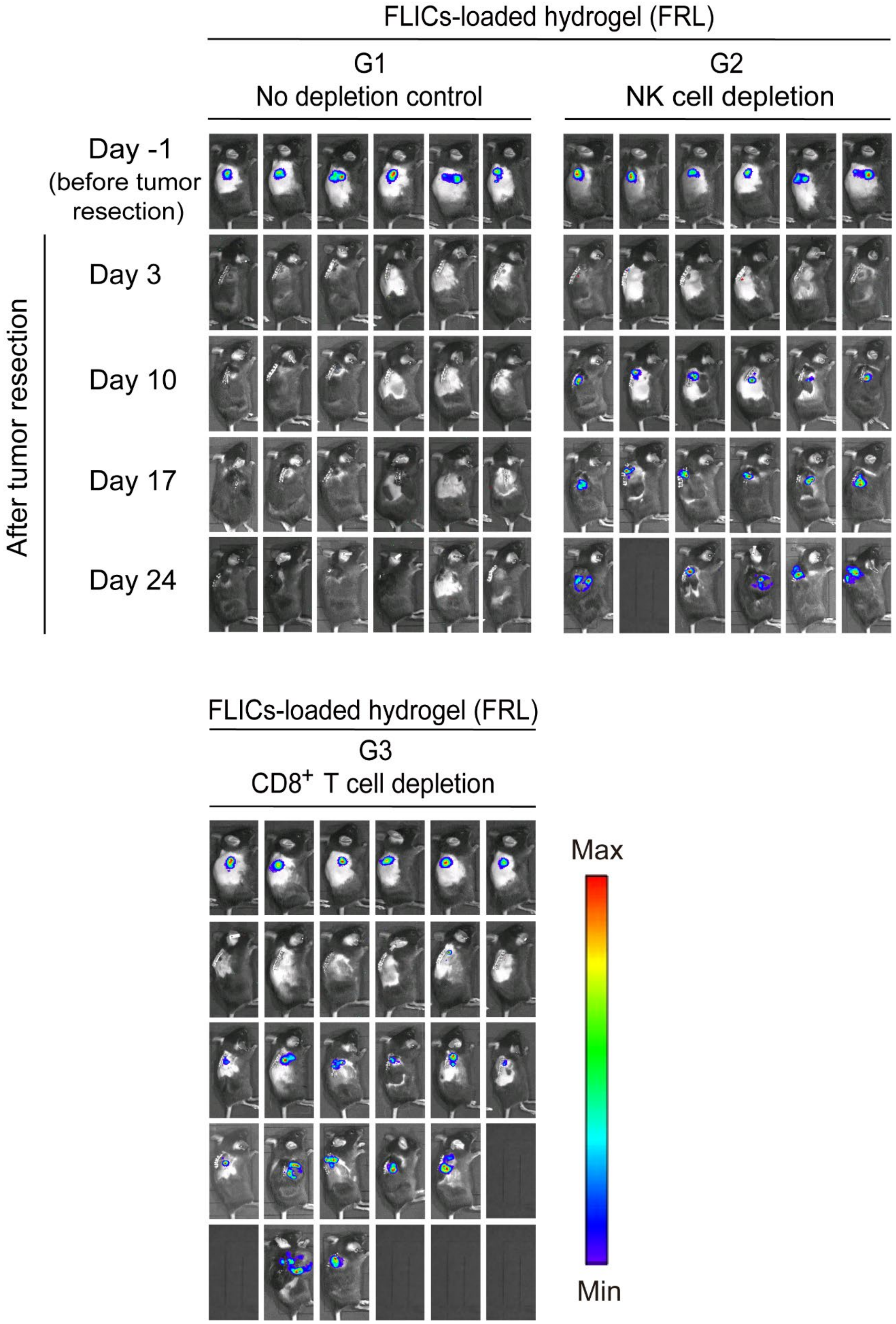

Supplementary Figure 16. Magnified images of Figure 5b.

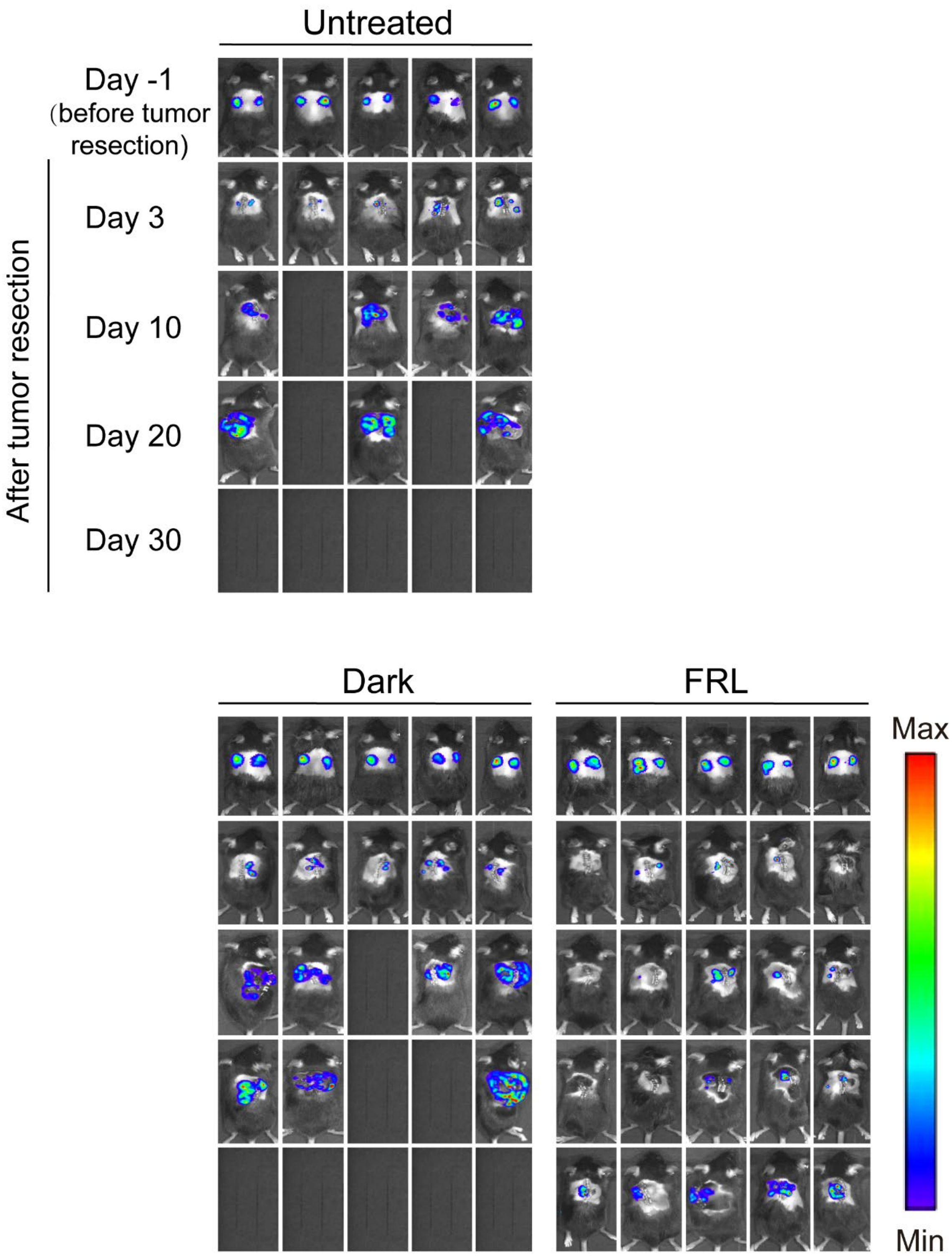

Supplementary Figure 17. Magnified images of Figure 6d.

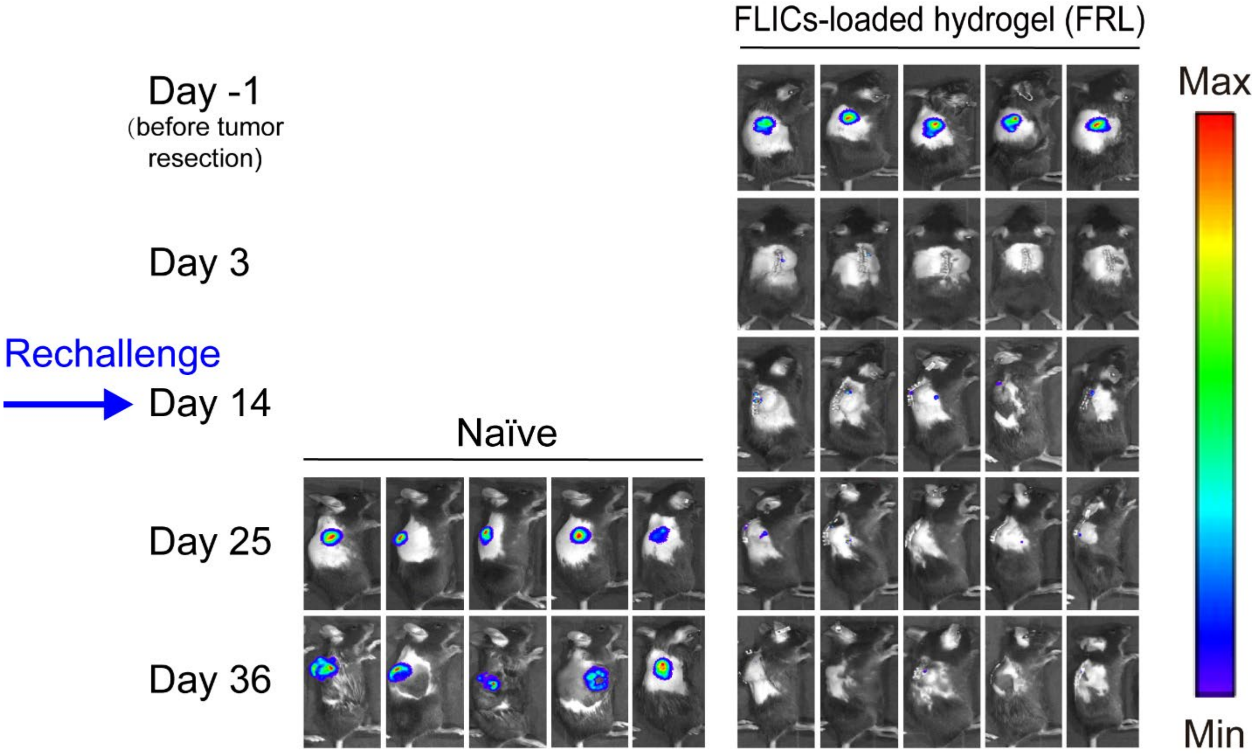

Supplementary Figure 18. Magnified images of Supplementary Figure 6a.

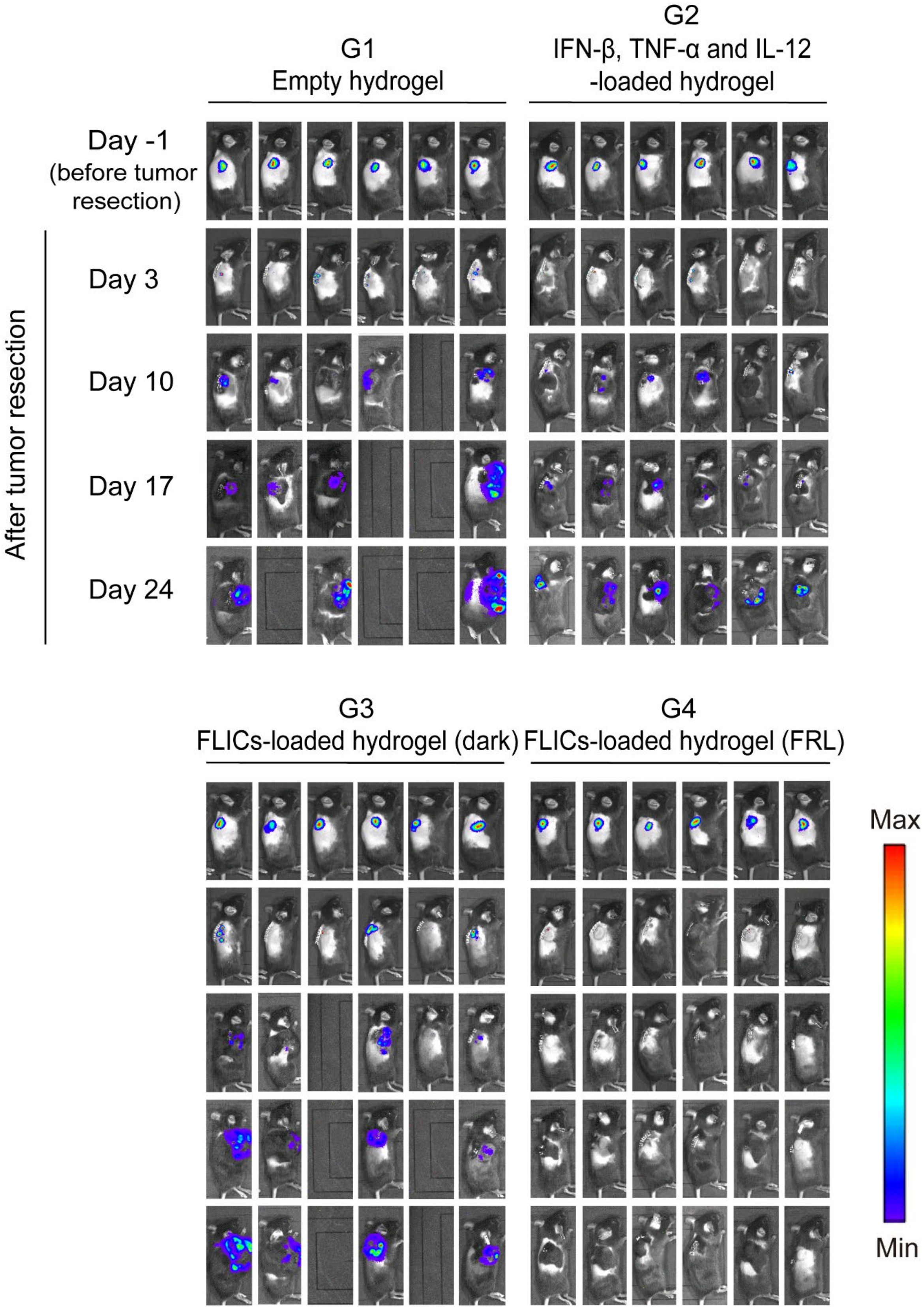

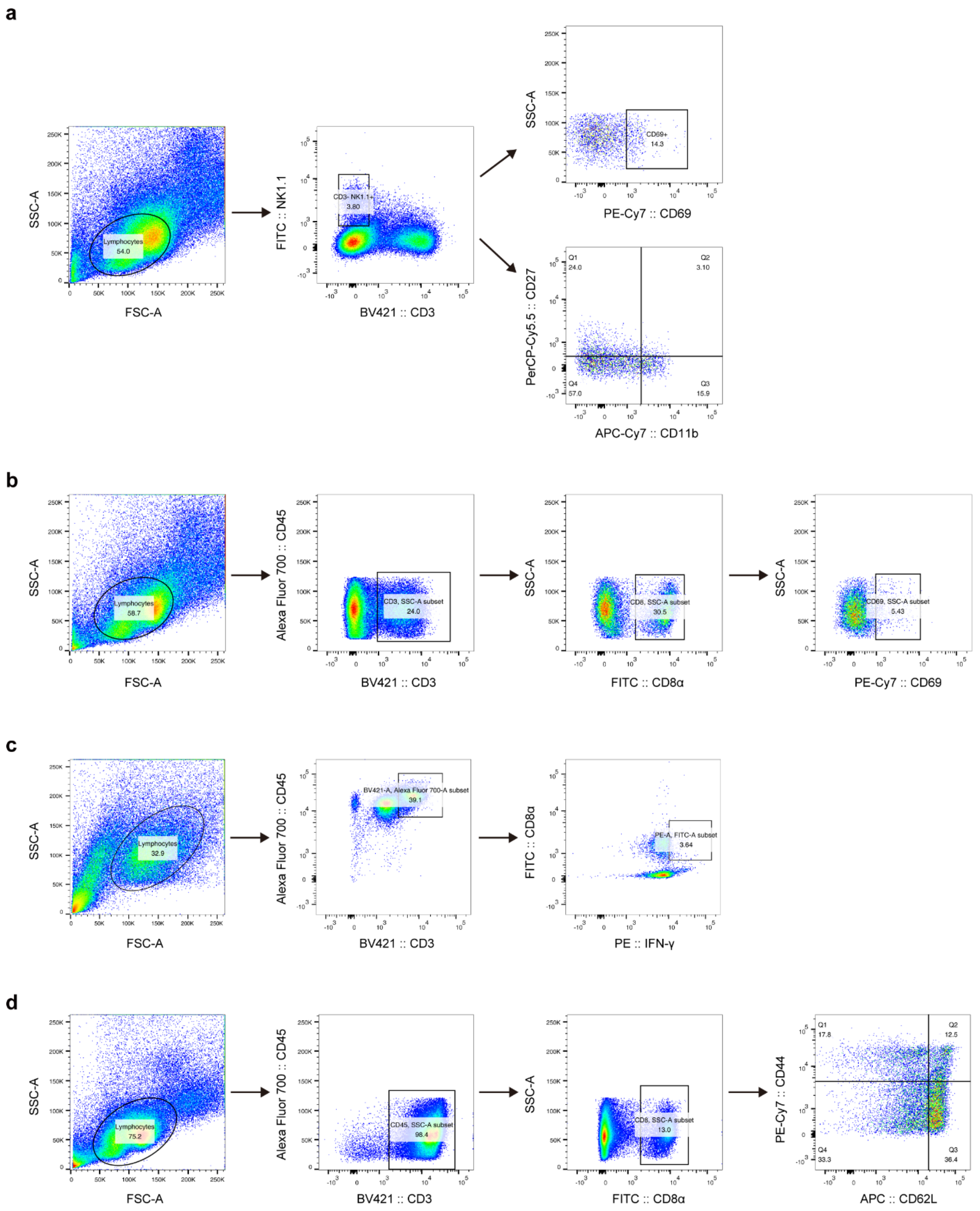

Supplementary Figure 19. Gating strategies of representative flow cytometry staining of NK cells and T cells. NK cells and T cells were gated on viability-dye negative cells to exclude dead cells, lymphogated on size to include lymphocytes, and doublets were excluded based on size (FSC) and granularity (SSC). Representative results of one independent experiment. (**a**) Expression of $CD3^-$ $NK1.1^+$ was used to define NK cells. Expression of $CD69^+$ and $CD11b^+CD27^+$ were used to define

activated NK cells. (**b**) Expression of $CD45^{+}CD3^{+}CD8^{+}$ was used to define total $CD8^{+}$ T cells. Expression of ($CD69^{+}$) $CD8^{+}$ was used to define activated $CD8^{+}$ T cells. (**c**) Expression of (IFN-γ^{+}) $CD8^{+}$ was used to define cytotoxic $CD8^{+}$ T cells. (**d**) Expression of ($CD44^{+}CD62L^{+}$) $CD8^{+}$ was used to define central memory T cells.

Supplementary Table 1. Plasmids designed and used in this study.

**Plasmid**	**Description and cloning strategy**	**Reference**
P_{hCMV}-SB100X	Constitutive mammalian P_{hCMV}-driven SB100X expression vector (P_{hCMV}-SB100X-pA).	Addgene (no. 34879)
pYH88	Constitutive mammalian stable expression vector for BphS, YhjH, PuroR and mCherry (ITR-P_{hCMV}-BphS-P2A-YhjH-P2A-p65-VP64-BldD-P2A-mCherry-pA::P_{mPGK}-PuroR-pA-ITR)	(1)
pYH428	Constitutive mammalian P_{FRL}-driven IFN-β, TNF-α, and IL-12 stable expression vector (ITR-P_{FRL}-IFN-β-P2A-TNF-α-P2A-IL-12-P2A-EGFP-pA::P_{mPGK}-ZeoR-pA-ITR)	This work
pYH500	Constitutive mammalian P_{hCMV}-driven IFN-β, TNF-α, and IL-12 stable expression vector (ITR-P_{hCMV}- IFN-β-P2A-TNF-α-P2A-IL-12-P2A-EGFP-pA::P_{mPGK}-ZeoR-pA-ITR)	This work

Abbreviations: BphS, engineered bacterial diguanylate cyclase; **BldD**, *Streptomyces coelicolor* transcription factor regulating aerial hyphae formation; **EGFP**, enhanced green fluorescent protein; **FRL**, far-red light; **IFN-β**, interferon beta; **IL-12**, interlukine-12; **ITR**, inverted terminal repeat; **mCherry**, mushroom Coral red fluorescence protein; **pA**, polyadenylation signal; **P_{FRL}**, FRL-v2-specific chimeric promoter; **P_{hCMV}**, human cytomegalovirus immediate early promoter; **P_{mPGK}**, mouse phosphoglycerate kinase gene promoter; **PuroR**, gene product that confers puromycin resistance to mammalian cells; **P2A**, picornavirus-derived self-cleaving peptide engineered for bicistronic gene expression in mammalian cells; **p65**, 65k Da transactivator subunit of NF-κB; **SB100X**, Sleeping Beauty transposase; **TNF-α**, tumor necrosis factor-α; **VP64**, tetrameric core of herpes simplex virus-derived transactivation domain; **YhjH**, bacterial c-di-GMP phosphodiesterase; **ZeoR**, gene product that confers zeocin resistance to mammalian cells.

Supplementary Table 2. Amino acids or DNA sequence information

1. BphS-P2A-YhjH

BphS	MARGCLMTISGGTFDPSICEMEPIATPGAIQPHGALMTARADSGRVAHA
P2A	SVNLGEILGLPAASVLGAPIGEVIGRVNEILLREARRSGSETPETIGSF
YhjH	RRSDGQLLHLHAFQSGDYMCLDIEPVRDEDGRLPPGARQSVIETFSSAM
	TQVELCELAVHGLQLVLGYDRVMAYRFGADGHGEVIAERRRQDLEPYLG
	LHYPASDIPQIARALYLRQRVGAIADACYRPVPLLGHPELDDGKPLDLT
	HSSLRSVSPVHLDYMQNMNTAASLTIGLADGDRLWGMLVCHNTTPRIAG
	PEWRAAAGMIGQVVSLLLSRLGEVENAAETLARQSTLSTLVERLSTGDT
	LAAAFVAADQLILDLVGASAAVVRLAGQELHFGRTPPVDAMQKVLDSLG
	RPSPLEVLSLDDVTLRHPELPELLAAGSGILLLPLTSGDGDLIAWFRPE
	HVQTITWGGNPAEHGTWNPATQRMRPRASFDAWKETVTGRSLPWTSAER
	NCARELGEAIAAEMAQRTRAEELERVAMVDSLTRLWNRLGIETLLKREW
	EYATRKNSPISIVMIDFDNFKQINDQHGHLVGDEVLQGSARLIISVLAS
	YDILGRWGGDEFMLILPGSGREQTAVLLERIQATIAQNPVPTSAGPMAI
	SLSMGGVSVFTNQGEALQYWVEQADNQLMKVKRLGKGNFQLAEYHHHHH
	HGSGATNFSLLKQAGDVEENPGPSGIRQVIQRISNPEASIESLQERRFWLQ
	CERAYTWQPIYQTCGRLMAVELLTVVTHPLNPSQRLPPDRYFTEITVSH
	RMEVVKEQIDLLAQKADFFIEHGLLASVNIDGPTLIALRQQPKILRQIE
	RLPWLRFELVEHIRLPKDSTFASMCEFGPLWLDDFGTGMANFSALSEVR
	YDYIKIARELFVMLRQSPEGRTLFSQLLHLMNRYCRGVIVEGVETPEEW
	RDVQNSPAFAAQGWFLSRPAPIETLNTAVLAL

2. p65-VP64-Linker-BldD

p65	MPSGQISNQALALAPSSAPVLAQTMVPSSAMVPLAQPPAPAPVLTPGPP
VP64	QSLMGSGRADALDDFDLDMLGSDALDDFDLDMLGSDALDDFDLDMLGSD
Linker	ALDDFDLDMLINASGSGGGGDVMASPKKKRKVEASSSEYAKQLGAKLRA
BldD	IRTQQGLSLHGVEEKSQGRWKAVVVGSYERGDRAVTVQRLAELADFYGV
	PVQELLPGTTPGGAAEPPPKLVLDLERLAHVPQEKAGPLQRYAATIQSQ
	RGDYNGKVLSIRQDDLRTLAVIYDQSPSVLTEQLISWGVLDADARRAVA
	HEEN

3. P_{FRL}: pA-3×whiG-$P_{hCMVmin}$

pA	CAGACATGATAAGATACATTGATGAGTTTGGACAAACCACAACTAGAAT
3×whiG	GCAGTGAAAAAAATGCTTTATTTGTGAAATTTGTGATGCTATTGCTTTA
$P_{hCMVmin}$	TTTGTAACCATTATAAGCTGCAATAAACAAGTTAACAACAACAATTGCA
	TTCATTTTATGTTTCAGGTTCAGGGGGAGGTGTGGGAGGTTTTTTAAAC
	CTCACGCTACGCTCACTCACGCTACGCTCACTCACGCTACGCTCACCTG
	CAGGTCGAGCTCGGTACCCGGGTCGAGTAGGCGTGTACGGTGGGAGGCC
	TATATAAGCAGAGCTCGTTTAGTGAACCGTCAGATCGCCTGGAGACGCC
	ATCCACGCTGTTTTGACCTCCATAGAAGACACCGGGACCGATCCAGCCT

CCGCG

4. IFN-β-P2A-signal peptide-TNF-α-P2A-IL-12

IFN-β<br>P2A<br>Signal peptide<br>TNF-α<br>P2A<br>IL-12	MNNRWILHAAFLLCFSTTALSINYKQLQLQERTNIRKCQELLEQLNGKI<br>NLTYRADFKIPMEMTEKMQKSYTAFAIQEMLQNVFLVFRNNFSSTGWNE<br>TIVVRLLDELHQQTVFLKTVLEEKQEERLTWEMSSTALHLKSYYWRVQR<br>YLKLMKYNSYAWMVVRAEIFRNFLIIRRLTRNFQNGSGATNFSLLKQAG<br>DVEENPGPMKIILWLCVFGLFLATLFPISWQMPVESGLSSEDSASSESF<br>AKRIKRHGLRSSSQNSSDKPVAHVVANHQVEEQLEWLSQRANALLANGM<br>DLKDNQLVVPADGLYLVYSQVLFKGQGCPDYVLLTHTVSRFAISYQEKV<br>NLLSAVKSPCPKDTPEGAELKPWYEPIYLGGVFQLEKGDQLSAEVNLPK<br>YLDFAESGQVYFGVIALDIGSGATNFSLLKQAGDVEENPGPSGCPQKLT<br>ISWFAIVLLVSPLMAMWELEKDVYVVEVDWTPDAPGETVNLTCDTPEED<br>DITWTSDQRHGVIGSGKTLTITVKEFLDAGQYTCHKGGETLSHSHLLLH<br>KKENGIWSTEILKNFKNKTFLKCEAPNYSGRFTCSWLVQRNMDLKFNIK<br>SSSSSPDSRAVTCGMASLSAEKVTLDQRDYEKYSVSCQEDVTCPTAEET<br>LPIELALEARQQNKYENYSTSFFIRDIIKPDPPKNLQMKPLKNSQVEVS<br>WEYPDSWSTPHSYFSLKFFVRIQRKKEKMKETEEGCNQKGAFLVEKTST<br>EVQCKGGNVCVQAQDRYYNSSCSKWACVPCRVRSGGGGSGGGGSGGGLA<br>SGGSMVSVPTASPSASSSSSQCRSSMCQSRYLLFLATLALLNHLSLARV<br>IPVSGPARCLSQSRNLLKTTDDMVKTAREKLKHYSCTAEDIDHEDITRD<br>QTSTLKTCLPLELHKNESCLATRETSSTTRGSCLPPQKTSLMMTLCLGS<br>IYEDLKMYQTEFQAINAALQNHNHQQIILDKGMLVAIDELMQSLNHNGE<br>TLRQKPPVGEADPYRVKMKLCILLHAFSTRVVTINRVMGYLSSA

Supplementary References

1. Shao, J. et al. Smartphone-controlled optogenetically engineered cells enable semiautomatic glucose homeostasis in diabetic mice. *Sci. Transl. Med.* **9**, eaal2298 (2017).
