# Supplementary material for: Optogenetic-controlled immunotherapeutic designer cells for post-surgical cancer immunotherapy
Source: Nat Commun. 2022 Oct 26;13:6357. doi: 10.1038/s41467-022-33891-9 (PMC9605972; doi:10.1038/s41467-022-33891-9)
Supplement: Supplementary file 3 — Reporting Summary [file 41467_2022_33891_MOESM3_ESM.pdf]

nature portfolio

Corresponding author(s): Haifeng Ye

Last updated by author(s): Oct 1, 2022

Reporting Summary

Nature Portfolio wishes to improve the reproducibility of the work that we publish. This form provides structure for consistency and transparency in reporting. For further information on Nature Portfolio policies, see our Editorial Policies and the Editorial Policy Checklist.

Statistics

For all statistical analyses, confirm that the following items are present in the figure legend, table legend, main text, or Methods section.

n/a	Confirmed	
☐	☒	The exact sample size (*n*) for each experimental group/condition, given as a discrete number and unit of measurement
☐	☒	A statement on whether measurements were taken from distinct samples or whether the same sample was measured repeatedly
☐	☒	The statistical test(s) used AND whether they are one- or two-sided<br>*Only common tests should be described solely by name; describe more complex techniques in the Methods section.*
☒	☐	A description of all covariates tested
☒	☐	A description of any assumptions or corrections, such as tests of normality and adjustment for multiple comparisons
☐	☒	A full description of the statistical parameters including central tendency (e.g. means) or other basic estimates (e.g. regression coefficient) AND variation (e.g. standard deviation) or associated estimates of uncertainty (e.g. confidence intervals)
☐	☒	For null hypothesis testing, the test statistic (e.g. *F*, *t*, *r*) with confidence intervals, effect sizes, degrees of freedom and *P* value noted<br>*Give P values as exact values whenever suitable.*
☒	☐	For Bayesian analysis, information on the choice of priors and Markov chain Monte Carlo settings
☒	☐	For hierarchical and complex designs, identification of the appropriate level for tests and full reporting of outcomes
☒	☐	Estimates of effect sizes (e.g. Cohen's *d*, Pearson's *r*), indicating how they were calculated

Our web collection on statistics for biologists contains articles on many of the points above.

Software and code

Policy information about availability of computer code

Data collection	Bioluminescence images of the mice were obtained using IVIS Lumina II in vivo imaging system (Perkin Elmer, USA).<br>Flow cytometry data were collected using the LSRFortessaTM Flow Cytometer (BD Biosciences).<br>Cytokines (IFN-β, TNF-α, and IL-12) in the cell culture supernatant were quantified using a Synergy H1 hybrid multi-mode microplate reader with Gen5 software (version: 2.04).<br>Cytokines in mouse plasma were quantified using the LSRFortessaTM Flow Cytometer (BD Biosciences, BD FACSDiva Software Diva8.0.2.).<br>Tumor tissue section imaging were obtained on an inverted fluorescence microscope (Leica DMI8, Wetzlar, Germany).
Data analysis	We used GraphPad (version 6) to perform statistical analysis.<br>Bioluminescence values of the mice were analyzed using Living Image® 4.3.1 software.<br>We used FlowJo software Version 10 to analyse Flow cytometry data.

For manuscripts utilizing custom algorithms or software that are central to the research but not yet described in published literature, software must be made available to editors and reviewers. We strongly encourage code deposition in a community repository (e.g. GitHub). See the Nature Portfolio guidelines for submitting code & software for further information.

Data

Policy information about availability of data

All manuscripts must include a data availability statement. This statement should provide the following information, where applicable:

- Accession codes, unique identifiers, or web links for publicly available datasets
- A description of any restrictions on data availability
- For clinical datasets or third party data, please ensure that the statement adheres to our policy

The data supporting the findings of this study are available within the article, in the Supplementary Information and in Source Data files. Source Data are provided with this paper.

Field-specific reporting

Please select the one below that is the best fit for your research. If you are not sure, read the appropriate sections before making your selection.

[x] Life sciences [ ] Behavioural & social sciences [ ] Ecological, evolutionary & environmental sciences

For a reference copy of the document with all sections, see nature.com/documents/nr-reporting-summary-flat.pdf

Life sciences study design

All studies must disclose on these points even when the disclosure is negative.

Sample size	No sample-size calculation was performed. Following standards of the field, sample sizes were estimated which were capable of yielding statistically significant. Typically, 4-6 mice per group were determined by the reproducibility of bioluminescence images.
Data exclusions	No data were excluded.
Replication	The number of independent experiments is specified in each figure legend, with at least 3 independent experiments, unless otherwise specified.
Randomization	Physical randomization. All the animals used were randomly selected from the delivered pool. Mice 6-8 weeks old were randomly selected from cages, divided into groups for the studies.
Blinding	The investigator is blinded to the group allocation and the sample at data collection.

Reporting for specific materials, systems and methods

We require information from authors about some types of materials, experimental systems and methods used in many studies. Here, indicate whether each material, system or method listed is relevant to your study. If you are not sure if a list item applies to your research, read the appropriate section before selecting a response.

Materials & experimental systems

n/a	Involved in the study	
[ ]	[x]	Antibodies
[ ]	[x]	Eukaryotic cell lines
[x]	[ ]	Palaeontology and archaeology
[ ]	[x]	Animals and other organisms
[x]	[ ]	Human research participants
[x]	[ ]	Clinical data
[x]	[ ]	Dual use research of concern

Methods

n/a	Involved in the study	
[x]	[ ]	ChIP-seq
[ ]	[x]	Flow cytometry
[x]	[ ]	MRI-based neuroimaging

Antibodies

Antibodies used

Alexa Fluor®-700 anti-mouse CD45 (catalog no. 103127, clone 30-F11,USA)
Brilliant Violet 421TM-anti-mouse CD3 (catalog no. 100228, clone 17A2,USA)
FITC-anti-mouse NK-1.1 (catalog no. 108705, clone PK136,USA)
PE/Cyanine7-anti-mouse CD69 (catalog no. 104511, clone H1.2F3,USA)
APC/Cyanine7-anti-mouse CD11b (catalog no. 101226, clone M1/70,USA)
PerCP/Cyanine5.5-anti-mouse CD27 (catalog no. 124213, clone LG.3A10,USA).
FITC-anti-mouse CD8α (catalog no. 100705, clone 53-6.7, USA)
PE/Cyanine7-anti-mouse CD44 (catalog no. 103029, clone IM7,USA)

APC-anti-mouse CD62L (catalog no. 104411, clone MEL-14,USA).
PE-anti-mouse IFN-γ (catalog no. 505808, clone XMG1.2,USA)
anti-mouse NK-1.1 (catalog no. 108760; Biolegend,USA)
anti-mouse CD8α (catalog no. 100764; Biolegend,USA)
anti-mouse CD8α antibody (Abcam #ab217344, UK)
Alexa Fluor 488 goat anti-rabbit immunoglobulin G antibody (Abcam #ab150077, UK)

Validation: All antibodies were validated for the specified application by respective manufacturer.

Eukaryotic cell lines

Policy information about cell lines

Cell line source(s)	Human mesenchymal stem cells (hMSC-TERT) were obtained from Professor Dr. Martin Fussenegger, Department of Biosystems Science and Engineering, ETH Zürich. The original source is from ATCC (SCRC-4000). B16F10-Luc (Luciferase tagged mouse B16F10 melanoma cancer cell line) and B16F10-OVA melanoma cells were obtained from Shanghai Sciencelight Biology Science & Technology Inc.
Authentication	None of the cell lines are authenticated.
Mycoplasma contamination	No mycoplasma contamination was detected for all cell lines.
Commonly misidentified lines (See ICLAC register)	No misidentified cell lines have been used in this study.

Animals and other organisms

Policy information about studies involving animals; ARRIVE guidelines recommended for reporting animal research

Laboratory animals	C57BL/6J mice (female, 6/8-week-old) were purchased from the ECNU (East China Normal University) Laboratory Animal Center.The female C57BL/6J wild-type mice (6-8 weeks old; ECNU Laboratory Animal Center) were kept in an animal house maintained at 22 ± 2° C, with a 12-hour light-dark cycle and free access to food and water.
Wild animals	None.
Field-collected samples	None.
Ethics oversight	The protocol involved in this study was approved by the ECNU Animal Care and Use Committee (protocol ID: m20200209)

Note that full information on the approval of the study protocol must also be provided in the manuscript.

Flow Cytometry

Plots

Confirm that:

- [x] The axis labels state the marker and fluorochrome used (e.g. CD4-FITC).
- [x] The axis scales are clearly visible. Include numbers along axes only for bottom left plot of group (a 'group' is an analysis of identical markers).
- [x] All plots are contour plots with outliers or pseudocolor plots.
- [x] A numerical value for number of cells or percentage (with statistics) is provided.

Methodology

Sample preparation	Collected cells were washed with cell staining buffer for 3 times, and then stained with specific antibodies. At least 5000 events were analyzed per sample.
Instrument	LSRFortessa™ Flow Cytometer (BD Biosciences)
Software	Data collection: BD FACSDiva Software Diva8.0.2<br>Data analysis: FlowJo 10
Cell population abundance	No sorting was conducted and single lymphocyte suspensions of spleens and peripheral blood were analyzed.
Gating strategy	Generally, cells wee first gated on FSC/SSC. Surface-antigen gating was performed on the live cell population.

- [x] Tick this box to confirm that a figure exemplifying the gating strategy is provided in the Supplementary Information.
